# On the Quest for Biomarkers: A Comprehensive Analysis of Modified Nucleosides in Ovarian Cancer Cell Lines

**DOI:** 10.3390/cells14090626

**Published:** 2025-04-22

**Authors:** Daniel A. Mohl, Simon Lagies, Alexander Lonzer, Simon P. Pfäffle, Philipp Groß, Moritz Benka, Markus Jäger, Matthias C. Huber, Stefan Günther, Dietmar A. Plattner, Ingolf Juhasz-Böss, Clara Backhaus, Bernd Kammerer

**Affiliations:** 1 Core Competence Metabolomics, Hilde-Mangold-Haus, University of Freiburg, 79104 Freiburg, Germany; 2 Institute of Organic Chemistry, University of Freiburg, 79104 Freiburg, Germany; 3 Pharmaceutical Bioinformatics, Institute of Pharmaceutical Sciences, University of Freiburg, 79104 Freiburg, Germany; 4 Department of Obstetrics & Gynecology, Medical Center-University of Freiburg, Hugstetter Str. 55, 79106 Freiburg, Germany; 5 Signaling Research Centre BIOSS, University of Freiburg, 79104 Freiburg, Germany; 6 Spemann Graduate School of Biology and Medicine (SGBM), University of Freiburg, 79104 Freiburg, Germany

**Keywords:** ovarian cancer, biomarkers, modified nucleosides, metabolic signature, mass spectrometry, HPLC

## Abstract

Ovarian carcinoma is a gynecological cancer with poor long-term survival rates when detected at advanced disease stages. Early symptoms are non-specific, and currently, there are no adequate strategies to identify this disease at an early stage when much higher survival rates can be expected. Ovarian carcinoma is a heterogeneous disease, with various histotypes originating from different cells and tissues, and is characterized by distinct somatic mutations, progression profiles, and treatment responses. Our study presents a targeted metabolomics approach, characterizing seven different ovarian (cancer-) cell lines according to their extracellular, intracellular, and RNA-derived modified nucleoside profiles. Moreover, these data were correlated with transcriptomics data to elucidate the underlying mechanisms. Modified nucleosides are excreted in higher amounts in cancer cell lines due to their altered DNA/RNA metabolism. This study shows that seven different ovarian cancer cell lines, representing different molecular subtypes, can be discriminated according to their specific nucleoside pattern. We suggest modified nucleosides as strong biomarker candidates for ovarian cancer with the potential for subtype-specific discrimination. Extracellular modified nucleosides have the highest potential in the distinguishing of cell lines between control cell lines and themselves, and represent the closest to a desirable, non-invasive biomarker, since they accumulate in blood and urine.

## 1. Introduction

Ovarian cancer (OC) is the deadliest cancer in women [1,2]. In 2023, more than 19,000 new cases were diagnosed in the United States and in 2020 more than 313,000 cases worldwide [3,4]. The majority of affected women are diagnosed at an advanced stage because ovarian cancer is usually asymptomatic or has nonspecific symptoms in the early stages [2,5]. The prognosis of ovarian carcinoma patients is related to the tumor stage at the time of diagnosis [6]. The earlier the diagnosis, the better the prognosis. For this reason, ovarian carcinoma has been widely studied. To date, no biomarker for ovarian cancer has demonstrated sufficient predictive or prognostic relevance to be incorporated into the clinical routine. CA-125 is used for monitoring treatment response or disease progression but has limited diagnostic value [7,8].

Thus, the question arises whether metabolomic screening of all women above a certain age is clinically meaningful.

Since early diagnosis of OC is challenging, leading to poor prognosis, it is crucial to identify novel biomarkers. Biomarkers can be raised at different levels along the central dogma of molecular biology: At the genomic, transcriptomic, proteomic, and metabolomic levels. The most prevalent biomarkers for OCs are raised and analyzed at the protein level, such as CA-125 [9,10]. Single molecules, proteins, genes, their transcripts, or a combination of these might also be used as biomarkers. An ideal biomarker should possess several key qualities: Accessibility: minimally to non-invasive biomarkers from blood or urine are preferred because of better patient compliance and reduced risks. Specificity: a biomarker should be specific to a particular disease, ensuring diagnosis with minimal false positives and the sensitivity to detect even small changes in a disease or early stages, reducing false negatives. It must provide reliable and reproducible results across different laboratories and conditions, and have clear clinical relevance, offering meaningful diagnostic, prognostic, or therapeutic insights. Ideally, it should be easy and cost-effective to measure using standardized techniques and should remain stable during sample collection, processing, and storage.

A hallmark of cancer is the metabolic reprogramming of cells, characterized by altered metabolic pathways, manifesting as changes in energy production and the utilization of amino acids, sugars, lipids, or normally rare biomolecules [11,12]. These alterations, expressed by the accumulation or decrease in specific metabolites, can be described as the metabolic signature of a certain cell line or cancer type, respectively. These signatures can serve as biomarkers and are much more specific than single parameters, such as single metabolites or proteins. Furthermore, these signatures are highly dynamic and sensitive to the slightest alterations in the environment or upstream processes, as metabolomics has the fastest response of all the omics techniques.

Few untargeted metabolomics studies in the past have identified modified nucleosides (hereinafter abbreviated as mNSs) as potential biomarkers, such as pseudouridine, 1-methyladenosine, 3-methyluridine, N4-acetylcytidine, 5′-Methylthioadenosine, and degradation products or biosynthesis intermediates, without focusing on a targeted analysis of mNS [13,14,15,16]. Moreover, it has been found that ovarian cancer tissues exhibit great differences regarding RNA modifications compared to normal ovarian tissue. This study analyzed the differences of 47 mNSs at the RNA-level compared to normal ovarian tissue [17]. Our study analyzed 60 modified nucleosides, which is, to the authors’ best knowledge, the greatest amount of analyzed nucleosides in regard to ovarian cancer. In other cancer types, such as breast, pancreas and kidney cancers, mNSs have already proven their suitability as biomarkers [18,19,20,21]. Moreover, intra- and extra-cellular mNSs, as well as RNA-derived mNSs, were analyzed. Nowadays, more than 150 different nucleoside modifications are known, ranging from simple modifications such as methylation or oxygen–sulfur exchanges to highly complex hyper modifications such as amino acid attachment or modification of the whole ring structure [22,23]. mNSs are considered metabolic waste products, since they cannot be recycled by salvage pathways due to the lack of specific enzymes responsible for their degradation, phosphorylation, and re-incorporation in RNA [20,24,25]. Moreover, nucleoside modifications occur mostly de novo, post-transcriptionally, which means that recycling possibilities are limited. That is the reason why mNS are excreted from cells and reach the bloodstream. They are concentrated in the urine, where they are prevalent in high concentrations [20,24,26]. This particular situation makes mNS interesting targets for non-invasive biomarker candidates.

The cell culture medium (CCM), reflecting the direct output of the exometabolome, is an important matrix for the study of biomarkers, since mNSs in the blood and urine are also exometabolomic excretions. The endometabolome refers to metabolites within cells, reflecting intracellular metabolic processes, whereas the exometabolome encompasses metabolites secreted or released into the extracellular environment, representing interactions with the surroundings.

Additionally, the role of mNSs as potential signaling or regulatory molecules remains uncertain. Moreover, mNSs may have additional signaling and regulatory roles in cancer development and progression. The role of mNSs can be in RNA as stabilizing or destabilizing components, altering translation efficiency either in m-, r-, or t-RNA [27,28,29,30]. Recent evidence suggests that this type of nucleoside modification benefits tumor growth and progression [31,32,33].

It has been demonstrated that N6-methyladenosine is a strong signaling molecule, stimulating purine receptors [24]. However, it is currently unknown whether other mNSs also play a role as signaling molecules.

The analysis of mNSs by high performance liquid chromatography (HPLC) coupled to triple quadrupole mass spectrometry (QqQ-MS) is the most common approach, due to its high sensitivity up to the single-digit femtomolar range; the high specificity is achieved by using dynamic multiple reaction monitoring and high-throughput screening [18,26,34,35]. Thus, in this work we applied a bottom-up biomarker discovery approach, starting from the cell model, which was analyzed via HPLC-QqQ-MS. This approach has the advantage of stepwise validation, which means that biomarkers identified in cell cultures can be further validated in more complex biological systems, such as animal models and human biological matrices. This stepwise approach ensures that the biomarkers are relevant and applicable in real-world clinical settings. Moreover, it is highly controlled and cost-effective, and indicators of mNSs as potential candidate biomarkers have already been made. Therefore, we consider this a valid approach to characterize ovarian cancer at the level of modified nucleosides, complementary to existing biomarkers.

### Aim of This Study

The aim of this study was to assess the metabolic signature of ovarian cancer via a multi-cell-line model at the level of excreted and endogenous free modified nucleosides, as well as RNA-derived modified nucleosides. Moreover, we employed a retrospective analysis of differential gene expression using publicly available datasets of ovarian cancer cell lines to uncover potential mechanistic insights. The goal of this assessment was to lay the foundation for a comprehensive analysis of patient-based biological matrices, such as blood, urine, ascites fluid, and (tumor-) tissue. By elucidating the metabolic nucleoside signature, pathophysiological mechanisms, therapeutic targets, and potential new biomarkers can be identified, which may contribute to an improved prognosis and diagnosis of ovarian cancers. For this purpose, an HPLC-QqQ-MS based analysis was conducted to compare and explore metabolic alterations between ten commonly used ovarian (cancer and a frequently used exemplary non-malignant control) cell lines.

## 2. Materials and Methods

### 2.1. Cell Culture

The investigated ovarian cancer and control cell lines, presented and described in Table 1, A2780, COV362, EFO21, EFO27, OAW42, SKOV3, and HOSE were cultured as described below. COV362 and SKOV3 cells were grown in DMEM high-glucose medium containing L-glutamine and pyruvate (Life Technologies, Darmstadt, Germany), supplemented with 10% fetal bovine serum (FBS) (Life Technologies, Darmstadt, Germany). OAW42 cells were grown in Gibco DMEM F12 medium (Life Technologies), containing GlutaMAX with Ham, supplemented with 10% fetal bovine Serum. The EFO21, EFO27, and HOSE 17.1 cell lines were grown in RPMI 1640 medium with GlutaMAX (Life Technologies), supplemented with 10% fetal bovine serum, and the A2780 cell line was grown in RPMI 1640 medium with GlutaMAX (Life Technologies) without FBS. All media were supplemented with 1% penicillin/streptomycin (Life Technologies, Darmstadt, Germany) and with 1% HEPES (Sigma-Aldrich/Merck KGaA, Darmstadt, Germany), and all cells were maintained in a humidified atmosphere containing 2.5% CO_2_ at 37 °C. Cell lines were originally purchased from CLS (CLS Cell Lines Service GmbH, Eppelheim, Germany: A2780, SKOV3, OAW42), Sigma-Aldrich (Merck KGaA, Darmstadt, Germany: COV362), or DSMZ (Leibniz-Institut DSMZ GmbH, Braunschweig, Germany: EFO21, EFO27), or kindly provided by Tilman Brummer, the Institute of Molecular Medicine and Cell Research, the University of Freiburg, Freiburg im Breisgau, Germany (HOSE 17.1, RRID: CVCL_7672) [36]. The authenticity of each cell line has been regularly confirmed via services from CLS (CLS Cell Lines Service GmbH, Eppelheim, Germany), DSMZ (Leibniz Institute, DSMZ-German Collection of Microorganisms and Cell Cultures GmbH, Braunschweig, Germany), and Multiplexion GmbH (Friedrichshafen, Germany).

Cells were cultured in 75 cm^2^ plastic flasks (Sarstedt AG & Co. KG, Nümbrecht, Germany) and grown at 37 °C/2.5% CO_2_. After reaching confluence, cells were washed with 8 mL 1× PBS (Life Technologies, Darmstadt, Germany), twice, and incubated with 3 mL 1× Trypsin/EDTA (Life Technologies, Darmstadt, Germany) at 37 °C/2.5% CO_2_. Trypsinization was stopped by adding medium with serum. For this study, cells were then seeded in 6-well plates (Sarstedt AG & Co. KG, Nümbrecht, Germany) and grown to 80% confluence at 37 °C/2.5% CO_2_ for subsequent preparation of cell culture medium, harvesting cells for analysis of modified nucleosides, and for total RNA preparation.

### 2.2. Preparation of the Cell Culture Medium

Right before cell harvest, 1.5 mL of cell culture medium was collected. The sample was centrifuged, and the supernatant was transferred into a new tube, which was snap-frozen in liquid nitrogen immediately. The samples were stored at −80 °C until the day of analysis. The samples were thawed at 4 °C overnight in the refrigerator and then vortexed for 30 s. Working on ice, 100 µL of cell culture medium was added to 900 µL of ice-cold precipitation solution (comprising acetonitrile:methanol (3:1, *v:v*), with 1 µg/mL isoguanosine, phenyl-β-D-glucopyranoside, ribitol, O-methyl-tyrosine, and heptadecanoic acid as internal standards). The sample was vortexed and for another 30 s and was centrifuged for 45 min at 20,000× *g* at 4 °C. A total of 100 µL of the supernatant was transferred into a new tube and the solvents were evaporated until complete dryness in a vacuum concentrator. The metabolite pellets were reconstituted in 100 µL of pure H_2_O. A total of 70 µL was transferred into LC vials and 20 µL of each sample was pooled to create a mixed quality control sample.

### 2.3. Preparation of the Cells for Targeted Analysis of Modified Nucleosides

The cells were grown until 80% confluency in 6-well plates (Sarstedt AG & Co. KG, Nümbrecht, Germany) with 3 mL of the respective cell culture medium. After counting and microscopical confirmation, the cells were harvested. The cell culture medium was discarded and immediately 2 mL of 0.9% of isotonic NaCl solution was added to wash away any remaining cell culture medium. This was repeated twice. A total of 1 mL of ice-cold precipitation solution was added to the six well quickly to quench the cells’ metabolism. The cells were scraped completely and transferred into a 2 mL tube, which was snap-frozen in liquid nitrogen. The samples were thawed vortexed and centrifuged (45 min, 20,000× *g*, 4 °C). Two aliquots of 300 µL were taken and evaporated in the vacuum concentrator. These pellets were stored at −80 °C until analysis. One aliquot was resuspended in 100 µL pure H_2_O and 100 µL CHCl_3_ and vortexed for 30 s, whereas the other was stored as backup. This step has the advantage of cleaning the sample and splitting non-polar and polar components, where 80 µL of the CHCl_3_ phase was transferred into a new tube, evaporated and stored at −80 °C for further analyses. A total of 70 µL of the aqueous phase, containing all nucleosides, was transferred into an LC vial for targeted analysis via HPLC triple quadrupole MS and 10 µL of each sample was pooled to create a mixed quality control sample.

### 2.4. RNA Extraction and Digestion

The total RNA of the cells has been extracted with the EURx GeneMATRIX Universal RNA Purification Kit for isolation of total RNA and miRNA from cell culture (Roboklon GmbH, Berlin, Germany/EURx Ltd., Gdansk, Poland) according to the manufacturer’s protocol and further details specified below. For the isolation of cellular RNA, grown and scraped cells from a 6-well plate were lysed with 1 mL RNA Extracol solution and mixed with the pipet. After 10 min of incubation at room temperature (RT) 200 µL of chloroform was added. The mixture was shaken vigorously for 15 s and centrifuged for 15 min at 4 °C and 12,000× *g*. After centrifugation, 400 µL of the upper aqueous phase was transferred to a fresh 2.0 mL tube and 480 µL of 100% ethanol solution was added. The mixture was shaken vigorously. The solution was transferred to RNA binding spin-column and centrifuged at RT for 1 min with 11,000× *g*. The flowthrough was discarded and 800 µL of wash solution (75% ethanol) was applied to the columns to centrifuge at RT for 1 min with 11,000× *g*. After discarding the flowthrough, this step was repeated. For removal of the residual buffer, the spin-column was centrifuged again at RT for 2 min with 12,000× *g* and then placed into a new 1.5 mL receiver tube. RNA was eluted with 40 µL of elution-solution (50 mm NH_4_(SO_4_)_2_) and was stored at −20 °C until use.

The RNA concentration of each sample was quantified using a portable UV/Vis spectrophotometer NanoPhotometer™ N60 (Implen GmbH, München, Germany). For pre-analysis normalization, 1 µg of RNA from each sample was evaporated in the vacuum concentrator and resuspended in 50 µL of 50 mm ammonium acetate buffer (pH 4.6) containing 100 nm isoguanosine as the internal standard. A total of 5 µL of Nuclease P1 solution (10.000 U/mL) was added (New England Biolabs GmbH, 65926 Frankfurt am Main, Germany) and incubated for 2 h at 37 °C at 1000 rpm. To cleave the phosphates, the samples were incubated with potato phosphatase (acidic) according to the manufacturers’ protocol (Sigma-Aldrich/Merck KGaA, Darmstadt, Germany). The enzymes were quenched and precipitated by adding 450 µL of pure MS-grade acetonitrile followed by vortexing (30 s) and centrifugation (20,000× *g*, 30 min). A total of 400 µL of each supernatants was transferred into a new tube and the solvent was removed in the vacuum concentrator. The nucleoside pellets were reconstituted in 80 µL pure H_2_O to achieve a concentration corresponding to 0.01 µg/µL RNA, and 70 µL was transferred into LC vials. Another 10 µL from each sample was pooled to create a mixed quality control sample.

### 2.5. Targeted Analysis of the Nucleosides

The nucleosides were separated by reversed-phase HPLC (Waters Acquity HSS T3, Waters GmbH, Eschborn, Germany; Agilent LC 1290 Infinity, Agilent Technologies, Waldbronn, Germany) coupled to a triple-quadrupole mass spectrometer (Agilent Technologies 6460 Triple Quad LC/MS, Waldbronn, Germany). Solvent A consisted of pure H_2_O with 0.1% formic acid and B consisted of MS-grade methanol with 0.1% formic acid. The gradient was 100% A for 5 min, to 30% B after 5 min, to 98% B after 10 min, holding 98% B for 5 min, switching back to 100% A and holding it for 5 min for re-equilibration of the column. The flow rate was set to 400 µL/min with a column temperature of 50 °C. A waste segment was added after 16 min, to protect the MS. For optimal target identification, the modified nucleosides were analyzed in dMRM-mode (dynamic multiple-reaction-monitoring mode). The dMRM table with the transitions and collision energies is shown in Supplementary Table S1. The following MS parameters were used: The gas temperature was set to 300 °C with a flow rate of 7 L/min. The sheath gas flow rate was 7 L/min at 350 °C. The nebulizer pressure was 50 psi. The mass spectrometer was operated with +4 kV capillary voltage and 500 V nozzle voltage. During analysis, the samples were kept at 4 °C. A total of 5 µL of each sample was injected in a randomized order with quality control samples injected regularly in between the samples. A list of the analyzed nucleosides is shown in Table 2.

### 2.6. Data Processing and Statistical Analysis

The LC-MS data were processed using Agilent MassHunter Qualitative Analysis (version B.07.00) and Agilent MassHunter Quantitative Analysis (version B07.01 SP2). The peak area of a nucleoside was divided by the internal standards’ (isoguanosine) peak area in each sample. For statistical analysis, Microsoft Excel 2016 and the R-based suite Metaboanalyst 6.0 were used [37]. The metabolomics datasets were range-scaled to make all features in the analysis equally important, to compare them within their response range. Images such as heat maps, PCAs, PLS-DAs were created with Metaboanalyst 6.0. One-way ANOVA was used for significance testing. For post-hoc analysis, Fisher’s LSD was used. The PLS-DA models were tested by 5-fold cross-validation using 8 components. The heat maps only show significantly altered nucleosides, as detected by ANOVA. The Euclidean distance measure and Ward’s clustering method have been used for the heat maps. ANOVA post-hoc analyses can be found in Supplementary Tables S2 and S3.

### 2.7. Transcriptome Analysis of a Set of Ovarian Cancer Cell Lines

RNA-Seq data were obtained from the Sequence Read Archive database for all the available ovarian cancer cell lines [38].

To improve data comparability and mitigate batch effects, the following criteria were applied when selecting the RNA-Seq datasets: Extraction of total RNA using TRIzol reagent; RNA library preparation following the Illumina protocol; paired-end sequencing; utilization of Illumina HiSeq Series sequencing platforms; and having at least two replicates. This resulted in the following cell lines and corresponding SRA accession numbers:

- A2780: SRX13261686, SRX13261687, SRX13261688
- SKOV3: SRX25507630, SRX25507631, SRX25507632
- EFO21: SRX7505973, SRX7505974, SRX7505975
- EFO27: SRR7151468, SRR8615287
- COV362: SRR8615949, SRR19537767
- iOSE: iOSE11 (SRR6376807, SRR6376809, SRR6376811); IOSE (SRR27406934, SRR27406935, SRR27406936)
- Ovary: SRX9417156, SRX9417157, SRX9417158, SRX9417159, SRX9417160, SRX9417161, SRX9417162

The acquired raw data were first subjected to quality control using FastQC (v0.12.1) [39]. Trimming was performed using Trimmomatic (v0.39) [40], with a quality score threshold of 20. The reads were then aligned to the human genome (hg19) using HISAT2 (v2.2.1) [41], and the count file was generated using HTSeq (v2.0.3) [42]. To reduce noise and ensure statistical robustness, genes with a corresponding total count of less than 20 were removed. Subsequently, differential gene expression analysis was performed using PyDESeq2 (v0.4.12) [43]. Due to the unavailability of HOSE cells in the SRA database, a set of not further specified ovary cells and iOSE cells was chosen as the control. After normalization, differential expression was determined based on a Walch test with a Benjamini–Hochberg correction for multiple testing. Significance was defined as adjusted *p*-value < 0.05 and |log2FC| > 1.

Using the Modomics database [22], enzymes involved in the metabolism of mNSs were identified. Finally, log2-transformed fold changes were compared to those of the excreted mNS.

**Table 1:** Properties, origin and subtype classification of the analyzed cell lines, according to the current literature and data collections, as referred. The comprehensive data presented within this table originate from the DepMap portal ** (DepMap: The Cancer Dependency Map Project at Broad Institute; https://depmap.org/portal/ (accessed on 11. September 2024)) and/or [44] * and OnkoKGTM, Cellosaurus, and references [45,46,47,48,49]. In this condensed table, we have listed the subtype classifications as reported by the respective sources, which are in part based on histopathological assessments. Nevertheless, specific mutational profiles—accessible in detail via resources such as the DepMap portal—may in some cases suggest an alternative subtype assignment. For instance, the molecular signature of EFO-27 is more consistent with a classification as clear cell ovarian carcinoma (CCOC). Used abbreviations are: SOC: serous ovarian cancer (OC), MOV: mucinous OC, EOV: endometrioid OC, HGSOC: high grade serous OC, CCOC: clear cell OC, uk: unknown. ^1^ Refers to a commonly used control cell line for ovarian cancer tissues of different origin with the described properties. ^2^ Refers to cell lines used as control cell lines in the transcriptomic analysis due to the unavailability of HOSE cells in the SRA database.

Cell Line	Abbrev.	Age	Disease (Cell Type ^1^)	Tumor Type/Origin	Subtypes */**	Genetic Alteration	Reference
A-2780	A2780	uk	ovarian endometrioid adenocarcinoma	primary/ovary	ENOC */**	RRAS2; SMARCA4; PIK3CA; MED12	[44,50,51,52,53]
COV362	COV362	uk	serous ovarian adenocarcinoma	metastatic/ pleural effusion	HGSOC */**	TP53; BRCA1	[44,50,52,53,54]
EFO-21	EFO—21	56	serous ovarian cystadenocarcinoma	metastatic/ ascites	CCOC * / SOC **	TP53	[44,50,53,55]
EFO-27	EFO27	36	mucinous ovarian adenocarcinoma	metastatic/ abdomen	ENOC * / MOV **	TP53; ERBB2	[44,50,55]
OAW-42	OAW42	46	serous ovarian cystadenocarcinoma	metastatic/ ascites	CCOC * / SOC **	PIK3CA	[44,50,52,53,56]
SK-OV-3	SKOV3	64	serous ovarian cystadenocarcinoma	metastatic/ ascites	CCOC * / SOC **	EP300; PIK3CA	[44,50,52,53,57]
HOSE 17-1 ^1^	HOSE	adult	^1^ ovarian surface epithelial cells	^1^ immortalized control cell line	-	HPV E6, E7 transformed	[58]
iOSE11 ^2^	iOSE	uk	ovarian surface epithelial cells	immortalized control cell line	-	TP49/TP52/TP53; TERT; hph (HygR)	[38,49]
IOSE ^2^	IOSE	uk	ovarian epithelial cells	immortalized control cell line	-	uk	[38]
Ovary ^2^	Ovary	uk	Ovary	control cell line	-	-	[38]

**Table 2:** List of all analyzed nucleosides with their corresponding abbreviation. Nucleosides are grouped by their parent base. In the further text, these abbreviations will be used to describe the nucleosides.

Nucleoside	Abbreviation	Nucleoside	Abbreviation
Adenosine	A	Guanosine	G
N6-Acetyladenosine	ac6A	Isoguanosine (ISTD)	ISOG
2′-O-Methyladenosine	Am	8-Hydroxy-2′-Desoxyguanosine	ho8dG
N6-Isopentenyladenosine	i6A	8-Hydroxyguanosine	ho8G
1-Methyladenosine	m1A	2′-O-Methylguanosine	Gm
1,2′-O-Dimethyladenosine	m1Am	1-Methylguanosine	m1G
N6,N6-Dimethyladenosine	m6,6A	N2,N2,7-Trimethylguanosine	m2,2,7G
N6-Methyladenosine	m6A	N2,N2-Dimethylguanosine	m2,2G
N6,2′-O-Dimethyladenosine	m6Am	N2,7-Dimethylguanosine	m2,7G
2-Methylthio-N6-Isopentenyladenosine	ms2i6A	N2-Methylguanosine	m2G
N6-Threonylcarbamoyladenosine	t6A	7-Methylguanosine	m7G
S-Adenosylhomocysteine	SAH	Uridine	U
S-Adenosylmethionine	SAM	3-(3-Amino-3-Carboxypropyl)Uridine	acp3U
5′-Methylthioadenosine	MTA	2′-O-Methyluridine	Um
N6-Succinyl Adenosine	N6SAR	5-Carboxymethyluridine	cm5U
Cytidine	C	5-Methyluridine	m5U
N4-Acetylcytidine	ac4C	5-Methoxycarbonylmethyluridine	mcm5U
2′-O-Methylcytidine	Cm	5-Methoxyuridine	mo5U
5-Formylcytidine	f5C	2-Thiouridine	s2U
5-Formyl-2′-O-Methylcytidin	f5Cm	2-Thio-2′-O-Methyluridine	s2Um
5-Hydroxymethylcytidine	hm5C	4-Thiouridine	s4U
N4,N4-Dimethylcytidine	m4,4C	Pseudouridine	Y
5-Methylcytidine	m5C	1-Methylpseudouridine	m1Y
5,2′-O-Dimethylcytidine	m5Cm	3-Methylpseudouridine	m3Y
3-Methylcytidine	m3C	Dihydrouridine	D
2-Thiocytidine	s2C	5-Hydroxyuridine	ho5U
Inosine	I	5-Methyldihydrouridine	m5D
2′-O-Methylinosine	Im	5-Methoxycarbonylmethyl-2-Thiouridine	mcm5s2U
1-Methylinosine	m1I	5-carbamoylmethyluridine	ncm5U
Others			
Xanthosine	X		
5-Aminoimidazole-4-Carboxamide Ribonucleotide	AICAR		
4-Demethylwyosine	imG-14		

## 3. Results and Discussion

### 3.1. Exo- and Endo-Metabolomic Analysis of the Cell Culture Medium and Ovarian Cell Lines

**Figure 1:**
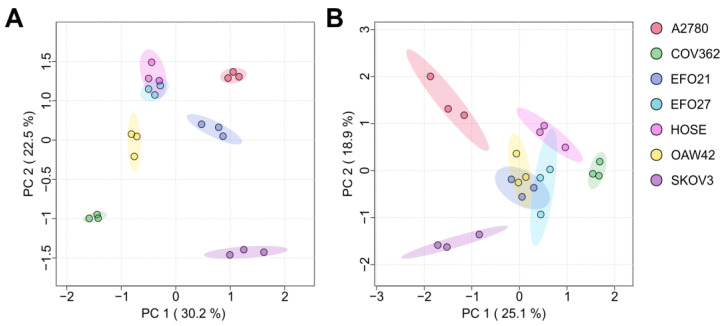
(**A**) Principal component analysis (PCA) of modified nucleosides in cell culture media. (**B**) Principal component analysis (PCA) of endogenous modified nucleosides in cells. PCA indicates differences between samples and groups. Shaded areas represent 95% confidence ellipses. Percentage of variance explained by each principal component reached 30.2% for PC1 and 22.5% for PC2 in (**A**), as wells as 25.1% for PC1 and 18.9% for PC2 in (**B**). Individual samples of a group are indicated by colored dots within the confidence ellipse. Each cell line, A2780, COV362, EFO21, EFO27, COV362, OAW42 and HOSE, was analyzed in biological triplicates (N = 3, for each cell line).

**Figure 2:**
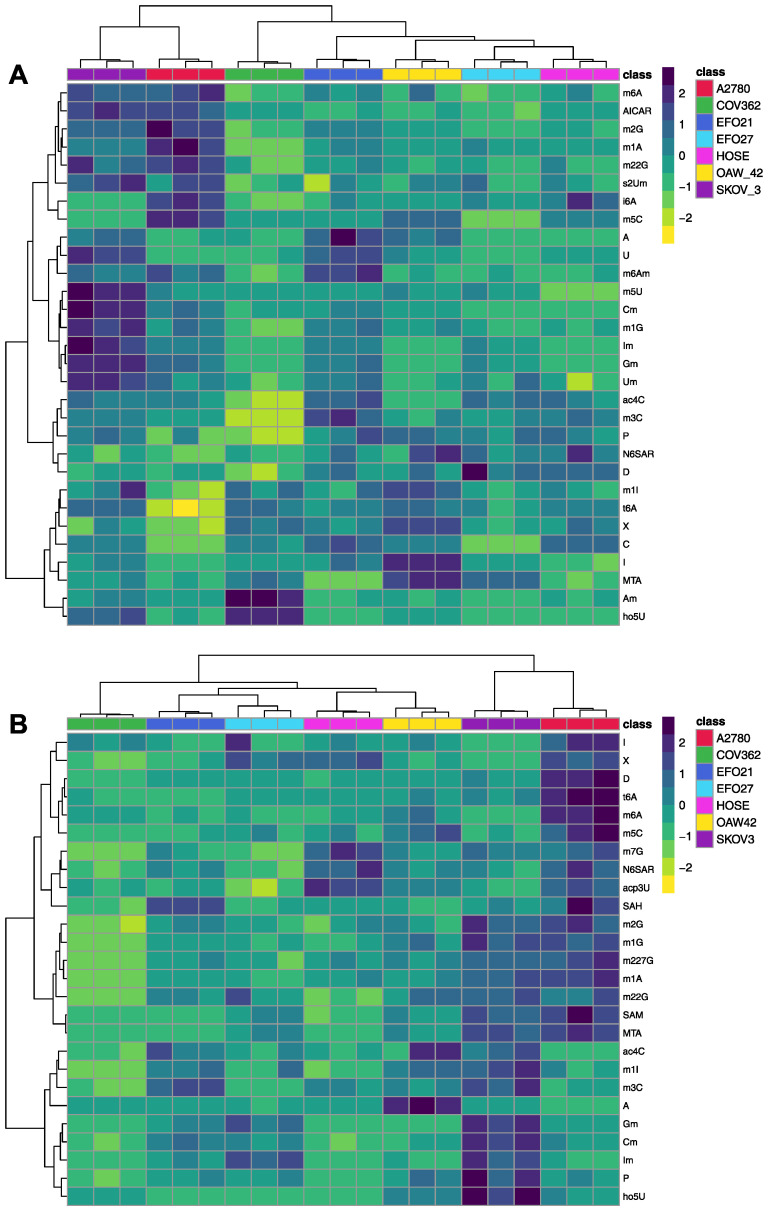
Clustered heat map representation of the exo- and endo-nucleoside analysis of the cell culture media (heat map (**A**)) and cells (heat map (**B**)). The color scale on the right indicates the relative concentrations in form of the range-scaled z-scores, where blue hues represent higher relative concentrations and yellow hues represent lower relative concentrations. Only significantly altered nucleosides are shown in both heat maps (one-way ANOVA, *p* < 0.05 after FDR). Each cell line is shown in triplicate (N = 3).

Cell culture medium (CCM) from cultured (cancer) cell lines can be considered as a representation of the tumor environment, displaying the exometabolome. In turn, the cells themselves represent a model of tumor tissue, which may be obtained during a biopsy and therefore display the endometabolome. Figure 1 shows the PCA (principal component analysis) plots of the exo- (Figure 1A) and endo-nucleosides (Figure 1B) analyzed in both the CCM and cells. All groups form distinct clusters, indicating that all tested cell lines differ significantly and can be distinguished based on their mNS profiles regarding intra- and extra-cellular nucleosides. Cell lines SKOV3, COV362, and A2780 are more distanced from the other cell lines (OAW42, EFO21, EFO27, and HOSE) in both plots, which means that their mNS profiles are more different than those of the others. Both analyses are also shown in the clustered heat maps in Figure 2. The cluster analysis reveals that cell lines A2780 and SKOV3 are more similar to each other than to the rest, regarding their intra- and extra-cellular mNS profiles.

This analysis highlights that every cancer cell line with its distinct genetic and transcriptomic characteristics represents an aspect and subtype of real cancer, illustrating the viability and dynamism of these genetic properties in relation to the metabolic output on the nucleoside level. Moreover, the analyzed cell lines are commonly used to describe different subtypes of ovarian cancers, as shown in Table 1. The PCA and the clustered heat map confirm that the mNS signature of these cell lines can specifically discriminate between ovarian cancer cell lines. As presented in Figure 2, cell lines A2780 and SKOV3 show the highest relative concentration of mNSs in both intra- and extra-cellular analysis. A2780 is one of the most commonly used cell lines for epithelial ovarian cancer but is often criticized for not being representative due to genomic and transcriptomic similarities to cancers other than OC [44,53]. This analysis supports this observation, since A2780 is more different from the other cell lines, as seen in the heat maps of Figure 2, featuring the highest intracellular levels of mNSs, such as I, X, D, t6A, and m6A. However, A2780 exhibits low concentrations of noted biomarker candidates P and ac4C, in contrast to SKOV3 [14,15]. Moreover, SKOV3 displays the highest levels of 2′-O-methylated mNSs intra- and extra-cellularly, like Cm, Gm, Im, Um, and s2Um. This modification is ubiquitous in eukaryotes and serves various functions, including stabilizing RNA species, as well as many still unknown roles [59]. Therefore, SKOV3 is believed to produce more stable RNAs for enhanced transcription, resulting in faster and efficient growth.

**Figure 3:**
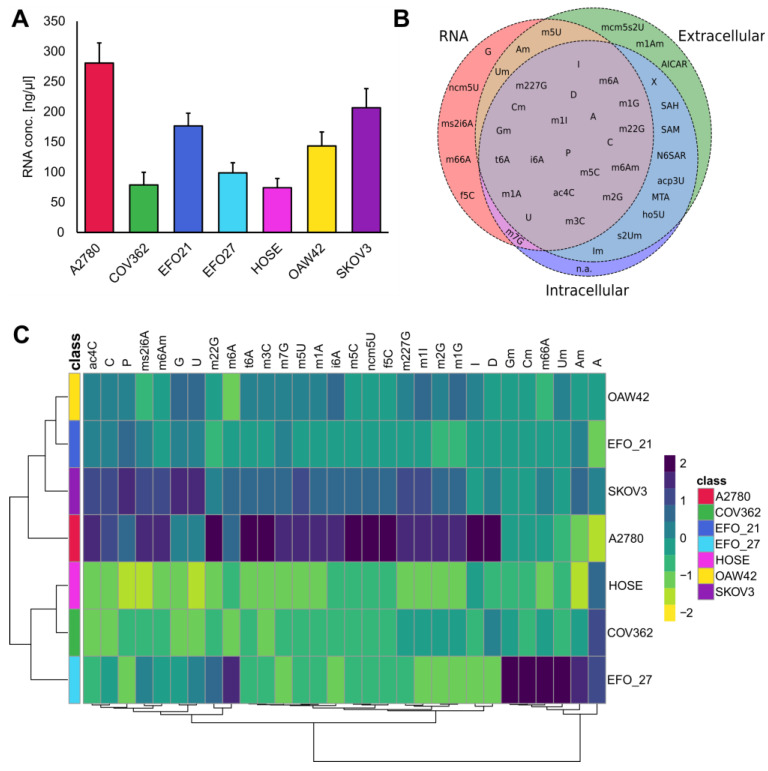
(**A**) RNA concentrations among all cell lines in ng/µl, ranging from 74 ng/ml (HOSE) to 280 ng/ml on average. Error bars indicate the standard deviation of each group. (**B**) Venn diagram showing modified nucleosides from RNA (red), extracellular (green), and intracellular (dark blue). Overlapping areas are color-coded as follows: RNA and intracellular overlap in pink, RNA and extracellular overlap in brown, extracellular and intracellular overlap in light blue, and the intersection of RNA, extracellular, and intracellular regions is shown in purple. (**C**) Heat map of the average relative concentration of the modified nucleosides of all groups derived from the RNA extracts. Only significantly altered nucleosides are shown (ANOVA, *p*-value < 0.05, FDR corrected). The color scale on the right indicates the relative concentrations in form of the range-scaled z-scores, where blue hues represent higher relative concentrations and yellow hues represent lower relative concentrations. (N = 3, every group shown as average).

Similarly, EFO27, which in contrast to A2780 and SKOV3, secretes considerably less mNSs and has lower intracellular concentrations of mNSs, but its RNA contains much more 2′-O-methylated mNSs (shown in Figure 3C). This may also correspond with more stable RNAs and more efficient transcription.

COV362 exhibits the lowest relative intracellular mNS levels but secretes the highest amounts of ho5U and Am. Notably, ho5U is known as a product of oxidative damage of DNA and RNA [60]. SKOV3 also secretes substantial amounts of ho5U and may be affected by oxidative DNA- and RNA-damage. However, COV362 has low intracellular levels of ho5U, while SKOV3 has high levels. These cell lines are therefore believed to be susceptible to oxidative nucleic acid damage, with COV362 excreting ho5U to the extracellular space. Moreover, ho5U has been reported to inhibit RNA and protein synthesis in bacteria, viruses, and tumor cells when introduced in the growth medium as a mononucleotide, indicating that cancer cell lines attempt to remove ho5U from the cell [61].

Recently, it has been demonstrated that mNS may also serve as signaling molecules, primarily in an extracellular context. Especially adenosine derivatives such as m6A and m1A have been identified as strong activators of the adenosine a3 receptor, stronger than A itself, resulting in increased ERK signaling and inflammation [24]. This makes m6A and other mNSs interesting candidates as potential signaling molecules that promote cancer growth in an autocrine manner.

The A2780 and SKOV3 cell lines secrete the highest amounts of m6A and m1A, from which their growth might profit. This effect could occur in vivo to benefit tumor growth and progression, as adenosine receptor a3 is overexpressed in many tumors [62].

These results, however, do not fully support the findings generated by genomic and transcriptomic analyses of these cell lines [44,50,53]. One would assume that cell lines labeled as endometrioid-, high-grade-serous-, clear cell ovarian cancer, etc. would cluster together since they have similar properties. However, our results could not confirm this expectation. It is important to note that many of these cell lines were established nearly half a century ago, and while genomic and transcriptomic data provide valuable insights, their classification may not always accurately reflect the complexity of primary human cancers. However, as this study focuses solely on cell lines without direct comparison to patient tumor samples, these models remain a valuable tool for investigation. However, nucleoside profiles that characterize the cell lines, are part of the metabolome, which is a much more sensitive and sensible indicator of the cellular phenotype, since features on the metabolomics level respond differently and much faster than those at the genomic, transcriptomic, or proteomic level [63,64]. Therefore, the mNS signature of a cell line does not necessarily correspond with the established assignment of a cell line. The profiling of mNSs adds another dimension of specific indicators that are able to separate cell line clusters depending on their “Modome”. Since the mNS profile provides a molecular characterization of cell lines, it has the potential to contribute to biomarker discovery. However, further studies are necessary to establish phenotypic correlations and validate its relevance for early detection and targeted treatment of ovarian cancer.

Moreover, RNA was extracted from all of the cell lines. The total RNA concentration for each cell line is shown in Figure 3A. To assess the relationships between free extracellular, intracellular, and RNA-incorporated mNSs, the extracted RNA was digested into single nucleosides, as described in the methods section. Additionally, we analyzed mNSs specific for a location, either intracellular, extracellular, or RNA-derived, as shown in Figure 3B. SAM, SAH, and MTA are not mNSs in the classical sense because they are not part of the RNA but play important roles in the methylation of nucleosides and are therefore not detectable in extracted RNA. All of them are part of the methionine/methylation cycle. The cell lines A2780 and SKOV3 demonstrate intracellular concentrations of SAM, SAH, and MTA, indicating an elevated methylation turnover. This is also reflected in (hyper-) methylated nucleoside species such as m227G, m22G, m7G, m5C, etc., which are higher in these cell lines. It is important to mention that altered methylation is a hallmark of cancer, indicating the cancerous metabolism of these cell lines as a representative model [65].

Hypermodified nucleosides like ms2i6A, t6A, i6A, and ncm5U are found more frequently in the RNA of the A2780 and SKOV3 cell lines. In mammals, ms2i6A has not been reported in cytoplasmic tRNAs. ms2i6A is only present in mitochondrial tRNAs and not in cytosolic tRNAs [66,67]. This observation may indicate increased mitochondrial activity in SKOV3 and A2780. Furthermore, ms2i6A is known to stabilize mRNA–tRNA interactions, enhancing transcription, of which SKOV3 and A2780 would profit [68]. t6A and i6A play roles in translation and ensure enhanced translation fidelity and speed [69]. The RNA of EFO27 exhibits remarkable concentrations of 2′-O-methylated mNSs compared to the other cell lines, such as Am, Um, Cm, and Gm. The 2′-O-methylation occurs in different RNA species and serves different purposes. 2′-O-methylation within tRNAs was shown to inhibit innate immune response, allowing tumors to evade detection by the immune system [70]. Due to increased levels of 2′-O-methylated mNSs, EFO27 might benefit from increased RNA stability [71].

AICAR and N6SAR are also not part of the RNA. AICAR is an intermediate in the synthesis of purines, whereas N6SAR is an intermediate of the purine nucleoside cycle, with both being indicators of an increased purine biosynthesis and turnover.

There are mNSs that are specific to their location, such as mcm5s2U, m1Am, and AICAR for the extracellular space. These nucleosides may have potential signaling functions. AICAR is potentially more highly concentrated in cancer cell lines compared to the controls, resulting from an increased purine biosynthesis [72]. Furthermore, it has been demonstrated that AICAR is an activator of AMP kinase, which, depending on the stage of cancer, either supports or inhibits tumor growth [73]. mcm5S2U is a highly conserved RNA modification in eukaryotes [74]. Due to its hypermodified form, the cells are unable to recycle it and must excrete it. The concentrations of mcm5S2U in the total RNA, as well as intracellularly, were below the limit of detection in our analysis.

RNA-specific RNA derived nucleosides include G, f5C, m66A, ms2i6A, and ncm5U. No mNSs were identified that occur exclusively in the intracellular space. It is important to mention that these mNSs are not necessarily connected to their respective loci, as they could be below the detection limit of our analysis.

Since each sample from the RNA-derived mNSs was adjusted to the same RNA concentration prior to analysis, we focus only on effects that are independent of RNA level, which is not consistent across all cell lines, as shown in Figure 3A. For better visibility, the group averages of the mNSs are displayed in Figure 3C. Cell lines with higher RNA levels cluster together, while those with lower RNA levels form distinct clusters, considering concentration effects were removed by normalization a priori.

These results demonstrate that ovarian cancer cell lines have distinct profiles of RNA-derived mNSs, which are able to separate each group in a clustering analysis. Moreover, these effects are independent of the RNA level, which vary among the cell lines.

### 3.2. The Ovarian Cancer Cell Line Versus the Ovarian Epithelial Cell Line: Cancer Versus Control

**Figure 4:**
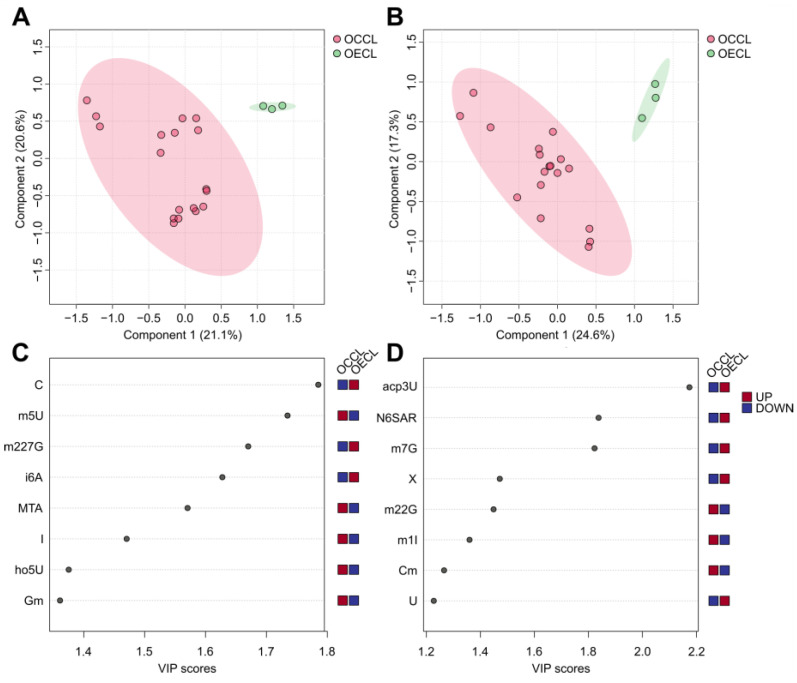
(**A**,**B**) The PLS-DA score plots of exo-nucleosides (**A**) and endo-nucleosides (**B**), which were grouped by ovarian epithelial cell line (OECL), representing the control group and ovarian cancer cell line (OCCL). The x- and y-axes express the variance for each group and data point in %. (**C**,**D**) The PLS-DA VIP score plot, which shows the eight top features (modified nucleosides) of component 1, which are most contributory for the clustering of the two groups OECL and OCCL. The red and blue color code implies higher or lower relative concentrations, respectively. (**C**) The PLS-DA VIP score plot for exo-nucleosides and (**D**) the PLS-DA VIP score plot for endo-nucleosides.

To create a situation analogues to a clinical study with patients and healthy control subjects, the cancer cell lines A2780, COV362, EFO21, EFO27, OAW42, and SKOV3 were merged into a new group, ovarian cancer cell line (OCCL), and HOSE, the control cell line, was labeled ovarian epithelial cell line (OECL). As the tissue-specific origin and subtypes of ovarian cancer (OC) are heterogeneous, which is reflected in the diverse subtypes of various OC cell lines used as in vitro model systems (see Table 1), the HOSE cell line (e.g., HOSE 17.1) represents a non-malignant ovarian tissue that is commonly employed as an exemplary control cell line [75]. To analyze the different nucleoside patterns between cancer and control, PLS-DA (partial-least squares discriminant analysis) was conducted. The scores plot created by PLS-DA is shown in Figure 4A,B. The exo-nucleoside analysis of the cell culture media is shown in Figure 4A and the endo-nucleoside analysis from the cells is shown in Figure 4B. The scores plots show that both, exo- and endo-nucleoside analysis form separate clusters for OCCL and OECL, which means that the pattern of extra- and intra-cellular modified nucleosides is capable of separating OCCL and OECL. To verify the quality of this model, 5-fold cross-validation with eight components was performed and Q^2^ was chosen as the quality assessment parameter. The model for exo-nucleosides yielded the best separation of OCCL and OECL with the use of seven components to reach maximum Q^2^ = 0.725, and the model for endo-nucleosides only needed three components for a maximum Q^2^ = 0.797. The cross-validation results are shown in Supplementary Figure S1A,B. Q^2^ values > 0.5 are considered high and represent good predictive ability of the model [76].

The top features of the extracellular mNSs are increased levels of m5U, MTA, ho5U, and Gm and reduced levels of C, m227G, and i6A, reaching VIP scores ranging from 1.2 to 2.2. A higher VIP score corresponds to a larger contribution to the separation of the OCCL and the OECL group. Regarding the analysis of patient samples such as urine and blood, special attention should be given to these nucleosides as potential biomarkers.

Intracellular mNSs increased in OCCL are m22G, m1I, and Cm. In contrast, mNSs increased in OECL are U, X, m7G, N6SAR, and acp3U. These candidates might be potential targets for analysis of patient-derived biopsies.

The cancer versus control model on the RNA-derived mNS level did not yield a proper discrimination of both groups (data shown in Supplementary Figure S2A,B). This model only achieved negative Q^2^ values, pointing out an over-fitted and unrepresentative model (shown in Supplementary Table S5). This finding emphasizes that extracellular mNSs are the most sensitive to discriminate ovarian cancer from control. In addition, the extracellular signature represents the non-invasive way of obtaining mNSs as biomarkers. Intracellular and RNA-derived mNS are unavoidably associated with invasive biopsies and resections of putative tumor tissue, increasing the risk for injuries and endangering patients.

It is also worth mentioning that the intra- and extra-cellular mNS patterns are quite different from each other, which is an indicator that nucleosides might fulfill different purposes dependent on their location. Further, the mNS profiles of the analyzed cell lines could be explained by differences in the number and type of nucleoside transporters, as well as in enzymes modifying and degrading mNSs.

Moreover, mNSs could perform as signaling molecules, activating pathways that benefit the growth of tumor cell lines, such as adenosine receptor activation that is known to increase inflammation or suppress the immune system, of which the tumor cells respond with increased growth and invasiveness, which has to be validated in further experiments [24,62,77]. Purine and pyrimidine receptors represent strong targets for these interactions, since both play important roles in the metabolic rewiring of cancer [78,79].

The presented data suggest that ovarian carcinoma, represented by six different cell lines in this study, has a specific modified nucleoside signature among themselves and the healthy control cell line HOSE. This signature is prevalent on all analyzed and possible origins of mNSs, with the clearest signature found extracellularly.

### 3.3. Transcriptome Analysis of the Ovarian Cancer Cell Lines Versus Control

To elucidate the mechanistic underpinnings of the higher abundance of mNSs in ovarian cancer, we performed a retrospective investigation of gene expression in the cell lines.

**Figure 5:**
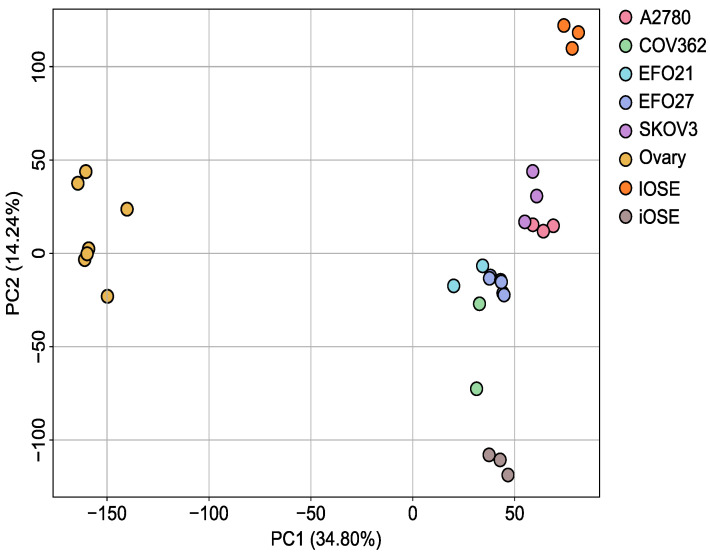
Principal component analysis (PCA) of cell lines based on gene expression patterns. PCA indicates differences between samples and groups. The percentage of variance explained by each principal component reached 34.8% for PC1 and 14.2% for PC2. Individual samples of a group are indicated by colored dots. Each cell line, A2780, EFO21, COV362, SKOV3, EFO27, Ovary, IOSE and iOSE, was analyzed in at least biological duplicates (N ≥ 2).

The PCA in Figure 5 shows a clear distinction between the ovarian cancer cell lines and the control cell lines, consisting of unspecified ovarian cells and immortalized OSE cells. However, the iOSE cells cluster closer to the cancer cell lines than to the ovary cells, most likely due to the immortalization process. We also used two sets of iOSE cells of different origin and immortalization strategy.

For the differential expression analysis, we focused on genes encoding nucleoside-modifying enzymes. The information for this mapping was obtained from the Modomics database [22]. Table 3 shows the log2-transformed fold change (log2FC) of significantly differentially expressed genes (DEGs) involved in the metabolism of mNSs. In addition to the respective enzymes, the corresponding mNSs are included.

The varying expression patterns of nucleoside-modifying enzymes may indicate different metabolic adaptations necessary for these cell lines. For example, the upregulation of AICDA in SKOV3 may indicate an increased need for stable RNA to support the rapid growth and chemoresistance of this cell line. TRDMT1 may serve as a biomarker for less aggressive subtypes such as EFO21, suggesting potential diagnostic applications.

**Figure 6:**
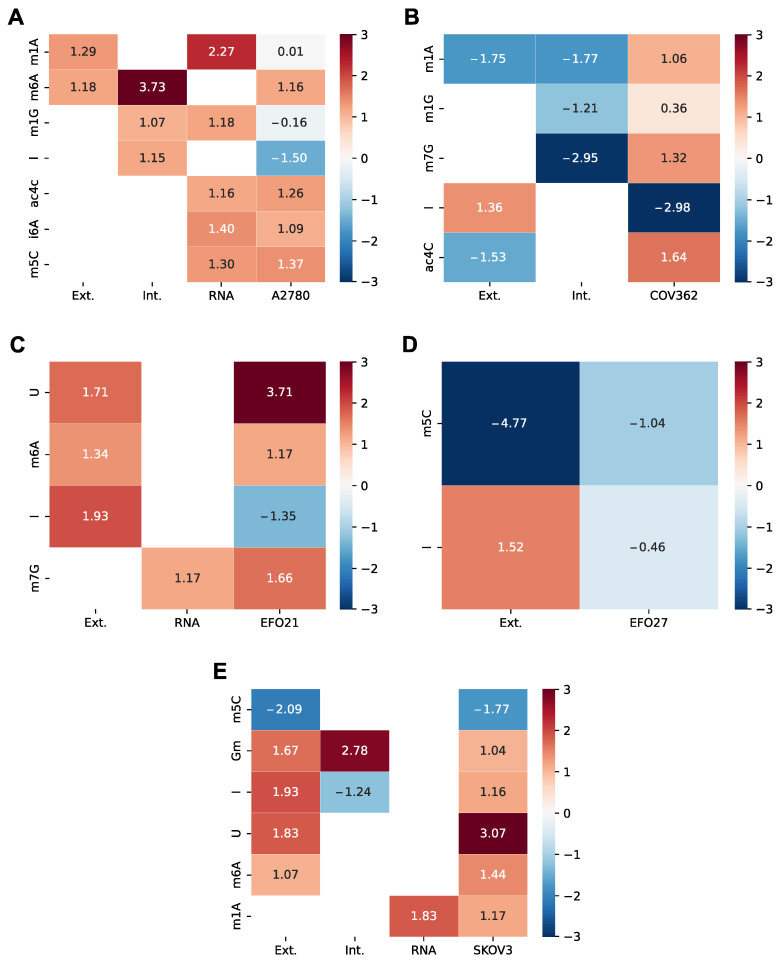
Heat map of log2FC of the mNSs and their nucleoside-modifying enzymes across the cell lines A2780 (**A**), COV362 (**B**), EFO21 (**C**), EFO27 (**D**), and SKOV3 (**E**), compared to the non-cancerous ovarian epithelial control cells. The rows represent individual mNSs, where the first two columns correspond to the measurement location (extracellular (Ext.), intracellular (Int.), or RNA-derived (RNA)) and the last column represents the transcriptome data and thus the gene expression of the nucleoside-modifying enzymes for the respective cell line. Significance is determined as *p* < 0.05 (FDR corrected) and |log2FC| > 1. White space indicates non-significance. For multiple enzymes contributing to the same mNS, mean log2FC values are displayed. The color scale on the right indicates relatively positive or negative log2FC.

The results in Table 3 show an increased presence of enzymes that convert nucleosides into mNSs, such as m1G, mcm5U, ac4C, and U in tumor cells. Simultaneously, there is a decrease of conversion enzymes that lead to m5C, mcm5s2U, hm6A, and I. This could be due to the accelerated metabolism of tumor cells, which implies an increased demand for mNSs and their degradation. Therefore, it would be expected that the log2FC of mNSs, either intracellular or extracellular or RNA-derived, would also be increased or decreased. We investigated this by comparing the log2FC of mNSs for each cell line and for each location with the log2FC of DEGs for each cell line for the corresponding mNS. The resulting heat maps are shown in Figure 6 for A2780 (Figure 6A), EFO21 (Figure 6B), EFO27 (Figure 6D), and SKOV3 (Figure 6E).

The heat map in Figure 6A shows that in the A2780 cell line, the log2FC of the nucleoside-modifying enzymes are elevated alongside the levels of the corresponding mNSs. The same trend can be seen for EFO21 and SKOV3 in Figure 6C and Figure 6E, respectively. However, this does not seem to be the case for I, which is overproduced in most measurements, but the corresponding enzymes are underexpressed. In SKOV3 (Figure 6E), however, I is overexpressed in the transcriptome data and also overproduced in the extracellular measurement, but not in the intracellular samples. Figure 6D,E show that the underexpression of TRDMT1 may lead to underproduction of m5C in EFO27 and SKOV3. In A2780 (Figure 6A), however, the overexpression of NSUN2 could lead to the increased levels of m5C in the RNA measurements. COV362 (Figure 6B) shows a different trend: an increase in the expression of the mNS-modifying enzyme is observed, while the corresponding mNS levels are decreased.

Therefore, with the retrospective transcriptome analysis, we could show a trend for some of the mNSs in that the increase or decrease of mNS levels is reflected in the transcriptome. However, we were not able to show this trend for all mNSs and not all to the same extent. This could be due to the different response times of the metabolome compared to the transcriptome and of course also to the use of data from a database, which is ultimately different from collecting data from the same source and under the same conditions as the mNS measurements.

Due to the unavailability of HOSE cells in the SRA database, we used alternative ovarian cell types as a baseline control. However, we acknowledge that most epithelial ovarian cancers—including high-grade serous carcinomas (originating from fallopian tube secretory cells) and clear cell carcinomas (arising from endometriotic lesions)—do not derive from the ovarian surface epithelium. This distinction is critical given the well-established cell-of-origin paradigm for ovarian cancer subtypes [80].

Despite this limitation, the mutual reinforcement of metabolomic and transcriptomic results across different control cell types underscores the discriminative power of the mNS approach in distinguishing cancer-associated profiles from non-malignant ovarian cells. Future studies could further improve comparability by incorporating fallopian tube epithelial cells (e.g., FT282 or FT194 lines) or endometriosis-derived models as controls, in line with the tissue-specific origins of ovarian cancer subtypes.

## 4. Conclusions

Our method offers a reliable approach for differentiating between pathological and metabolic states in ovarian cancer using a model system of seven distinct cell lines. This suggests that the method has potential for application in a clinical setting to screen for ovarian cancer (sub)types. The primary goal is to present a new biomarker on the modified nucleoside landscape, which can predict the (sub)type of ovarian cancer and may have implications for prognosis. This method can be applied to biological matrices such as blood and urine, which are collected during routine checkups. Moreover, ascites fluid and tumor tissues collected during resection surgery or biopsies can be analyzed similarly to map the mNS patterns, to determine the state and subtype of cancer. However, the heterogeneity of tumor samples may pose challenges for data interpretation, requiring further validation in patient-derived samples. The modified nucleoside landscape could enhance the characterization of ovarian cancer and advance personalized medicine. Distinct patterns of modified nucleosides in ovarian cancer could serve as specific biomarkers for different cancer subtypes, aiding in precise diagnosis. These profiles reflect tumor heterogeneity and indicate disease progression and metastasis, providing insights into the aggressiveness of the cancer. Distinct transcriptomic patterns observed across the cell lines further support the levels of modified nucleosides, highlighting a molecular trend that underscores the reliability of these biomarkers in predicting ovarian cancer subtypes and advancing personalized therapeutic approaches. Personalized biomarkers derived from nucleoside profiles enable tailored treatment plans and monitor treatment responses, offering early indications of effectiveness. Additionally, detecting modified nucleosides in body fluids allows for minimally invasive testing. This comprehensive molecular fingerprint aids in predicting outcomes and recurrence, ensuring precise and dynamic management of ovarian cancer. To further validate these findings and assess their transferability, a clinical study is planned to assess the stability and the clinical benefit of the nucleoside signatures observed in cell cultures when applied to patient-derived human samples.

## Figures and Tables

**Table 3:** log2FC of nucleoside-modifying enzymes across cell lines A2780, COV362, EFO21, EFO27, and SKOV3 against the control. Missing entries indicate no significant differential expression for that enzyme in the respective cell line. Significance is determined as *p* < 0.05 (FDR corrected) and |log2FC| > 1.

Enzyme	mNS	Log2FC A2780	Log2FC COV362	Log2FC EFO21	Log2FC EFO27	Log2FC SKOV3
ADARB1	I	−1.50	−2.91	−1.27	−1.32	
ADAT2	I				1.04	
ADAT3	I		−3.04	−1.43	−1.10	1.16
AICDA	U			3.71		3.07
ALKBH1	hm5C; f5C; f5Cm	1.29				
ALKBH8	nchm5U; mcm5U		1.22			
APOBEC1	U		2.01			
CTU1	mcm5s2U	−2.54	−2.31	−1.01	−2.16	
DIMT1	m6A	1.16		1.04		
FTO	hm6A; f6A	−1.11	−2.64	−1.07		
METTL1	m7G		1.32	1.66		
MRM1	Gm		−1.66			1.04
NAT10	ac4C	1.26	1.64			
NSUN2	m5C	1.37				
PUS1	Y	1.62				1.31
TRDMT1	m5C		−1.61	−2.91	−1.04	−1.77
TRMO	m6t6A				1.01	1.02
TRMT10A	m1G	1.08	2.17			
TRMT10B	m1G	−1.39	−2.17	−1.31	−1.50	
TRMT10C	m1G; m1A	1.22	1.06	1.03		
TRMT112	m6A; m2G; mcm5U		1.52	1.17		1.44
TRMT61A	m1A	−1.21				1.17

## Data Availability

MS data are available and can be found in Supplementary Table S4.

## Supplementary Materials

The following supporting information can be downloaded at: https://www.mdpi.com/article/10.3390/cells14090626/s1, Figure S1: 5-fold cross validation results of the PLS-DA Cancer vs. Control Model; Figure S2: PLS-DA Modell of ovarian cancer cell lines (OCCL) versus ovarian epithelial cell line (OECL) in RNA; Table S1: MRM transitions and parameters; Table S2: Cell culture medium ANOVA post-hoc; Table S3: Cells ANOVA post-hoc; Table S4: RNA ANOVA post-hoc; Table S5: MS data normalized; Table S6: 5-fold cross-validation results.
