# Supplementary material for: On the Quest for Biomarkers: A Comprehensive Analysis of Modified Nucleosides in Ovarian Cancer Cell Lines
Source: Cells. 2025 Apr 22;14(9):626. doi: 10.3390/cells14090626 (PMC12071701; doi:10.3390/cells14090626)
Supplement: Supplementary file 1 [file cells-14-00626-s001.zip › Supplementary_figures_S1_S2_and_table_S6.pdf]

On the Quest for Biomarkers: A Comprehensive Analysis of Modified Nucleosides in Ovarian Cancer Cell Lines – Supplementary

Daniel A. Mohl [1,2], Simon Lagies [1,2], Alexander Lonzer [3], Simon P. Pfäffle [3], Philipp Groß [4], Moritz Benka [1,2], Markus Jäger [4], Matthias C. Huber [4], Stefan Günther [3], Dietmar A. Plattner [2], Ingolf Juhasz-Böss [4], Clara Backhaus [4,*] and Bernd Kammerer [1,2,5,6*]

[1] Core Competence Metabolomics, Hilde-Mangold-Haus, University of Freiburg, 79104 Freiburg, Germany; daniel.mohl@ocbc.uni-freiburg.de (D.A.M.)
[2] Institute of Organic Chemistry, University of Freiburg, 79104 Freiburg, Germany
[3] Pharmaceutical Bioinformatics, Institute of Pharmaceutical Sciences, University of Freiburg, 79104 Freiburg, Germany
[4] Department of Obstetrics & Gynecology at the Medical Center-University of Freiburg, Hugstetter Str. 55, 79106, Freiburg, Germany.
[5] Signaling Research Centre BIOSS, University of Freiburg, 79104 Freiburg, Germany
[6] Spemann Graduate School of Biology and Medicine (SGBM), University of Freiburg, 79104 Freiburg, Germany
[*] Correspondence: Bernd Kammerer (BK) and Clara Backhaus (CB) share the correspondence for this article. Contact: bernd.kammerer@ocbc.uni-freiburg.de (BK); clara.backhaus@uniklinik-freiburg.de (CB)

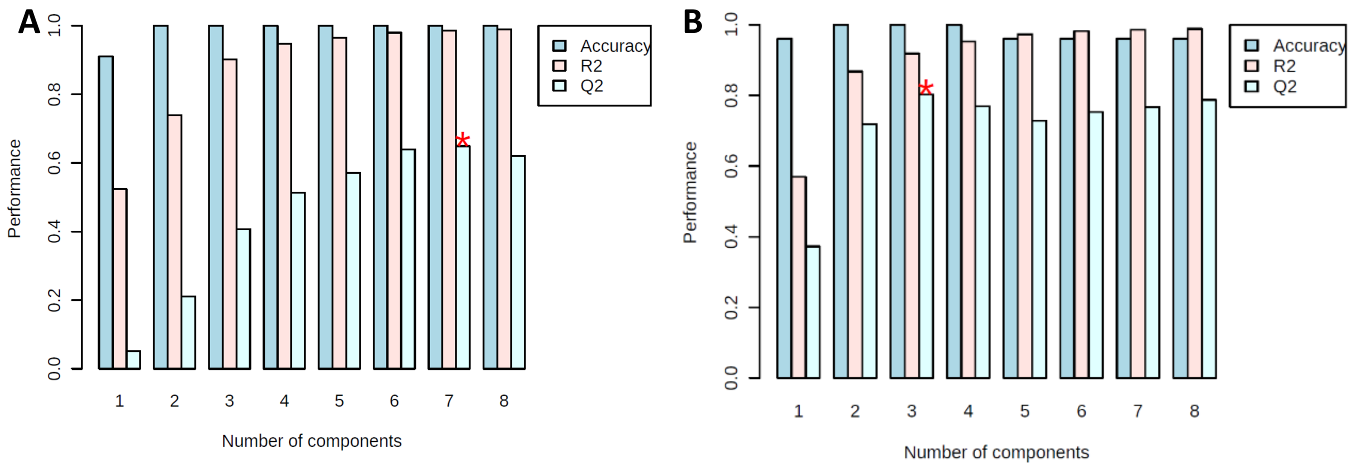

*Figure S1: 5-fold cross validation results of the PLS-DA Cancer vs. Control Model, with 8 components and Q^2 as measure of the performance. **A**: extracellular modified nucleosides. **B**: intracellular modified nucleosides. Highest Q^2 is marked with an asterisk.*

Table S6: 5-fold Cross validation Results of the Cancer versus Control model of RNA-derived modified nucleosides. With eight components.

measure	1 comps	2 comps	3 comps	4 comps	5 comps	6 comps	7 comps	8 comps
Accuracy	0.88	0.84	0.8	0.65333	0.54667	0.58667	0.62667	0.56
R2	0.22296	0.37029	0.44054	0.51846	0.58593	0.63941	0.74587	0.82151
Q2	-0.11787	-0.35706	-0.73216	-1.5457	-2.5094	-4.2407	-6.3603	-8.8756

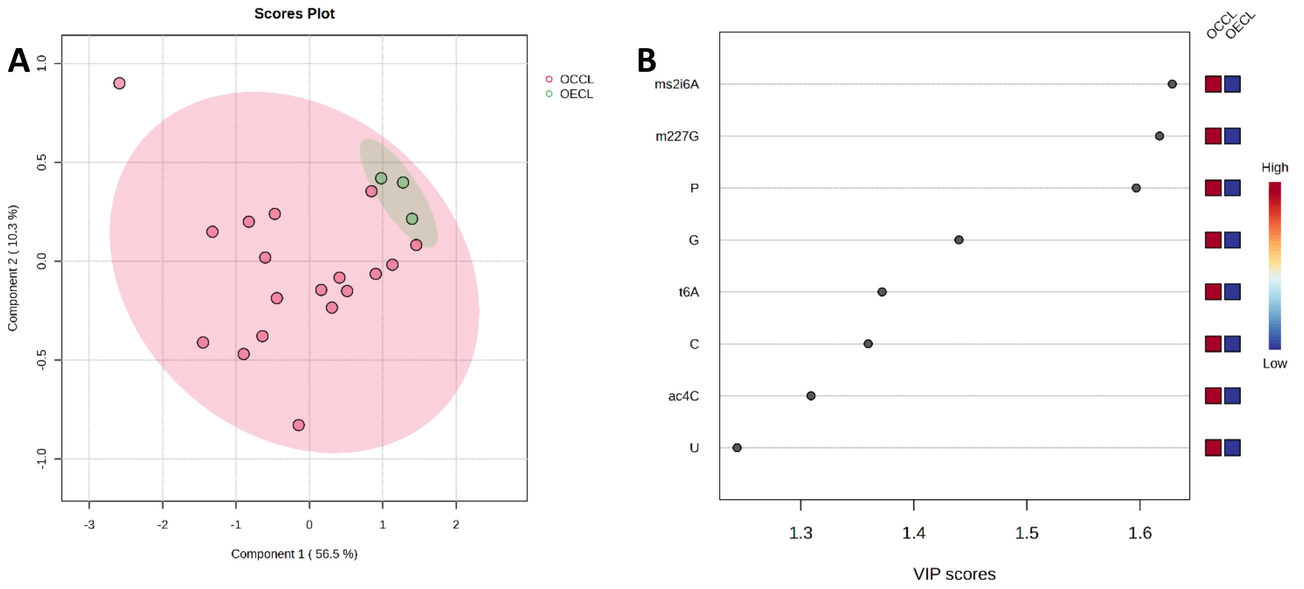

Figure S2: PLS-DA Modell of ovarian cancer cell lines (OCCL) versus ovarian epithelial cell line (OECL) in RNA. ***A****: PLS-DA scores plot and* ***B:*** *VIP-scores plot.*
